# Prevalence and clinical impact of *Streptococcus pneumoniae* nasopharyngeal carriage in solid organ transplant recipients

**DOI:** 10.1186/s12879-019-4321-8

**Published:** 2019-08-06

**Authors:** Cristina Roca-Oporto, Tania Cebrero-Cangueiro, María Luisa Gil-Marqués, Gema Labrador-Herrera, Younes Smani, Francisco Manuel González-Roncero, Luis Miguel Marín, Jerónimo Pachón, María Eugenia Pachón-Ibáñez, Elisa Cordero

**Affiliations:** 1 Clinical Unit of Infectious Diseases, Microbiology, and Preventive Medicine Infectious Diseases Research Group Institute of Biomedicine of Seville (IBiS), University of Seville/CSIC/University Hospital Virgen del Rocío, Seville, Spain; 2 Institute of Biomedicine of Seville (IBiS), University of Seville/CSIC/University Hospital Virgen del Rocio Seville, Seville, Spain; 3 Urology and Nephrology Unit, University Hospital Virgen del Rocío, Seville, Spain; 4 Clinical Unit of General Surgery, University Hospital Virgen del Rocío, Seville, Spain; 5 Department of Medicine, University of Seville, Seville, Spain

**Keywords:** *Streptococcus pneumoniae*, Solid organ transplant recipients and nasopharyngeal carriage

## Abstract

**Background:**

*S. pneumoniae* is the leading cause of community-acquired pneumonia in the solid organ transplant recipient (SOTR); nevertheless, the prevalence of colonization and of the colonizing/infecting serotypes has not been studied in this population. In this context, the aim of the present study was to describe the rate, characteristics, and clinical impact of *S. pneumoniae* nasopharyngeal carriage.

**Methods:**

A prospective observational cohort of Solid Organ Transplant recipients (SOTR) was held at the University Hospital Virgen del Rocío, Seville, Spain with the aim to evaluate the *S. pneumoniae* colonization and the serotype prevalence in SOTR. Two different pharyngeal swabs samples from 500 patients were included in two different seasonal periods winter and spring/summer. Optochin and bile solubility tests were performed for the isolation of thew strains. Antimicrobial susceptibility studies (MICs, mg/l) of levofloxacin, trimethoprim-sulfamethoxazole, penicillin, amoxicillin, cefotaxime, ceftriaxone, erythromycin, azithromycin and vancomycin for each isolate were determined by E-test strips. Capsular typing was done by sequential multiplex PCR reactions. A multivariate logistic regression analysis of factors potentially associated with pneumococcal nasopharyngeal carriage and disease was performed.

**Results:**

Twenty-six (5.6%) and fifteen (3.2%) patients were colonized in winter and spring/summer periods, respectively. Colonized SOT recipients compared to non-colonized patients were more frequently men (79.5% vs. 63.1%, *P* < 0.05) and cohabitated regularly with children (59% vs. 32.2%, *P* < 0.001). The most prevalent serotype in both studied periods was 35B. Forty-five percent of total isolates were included in the pneumococcal vaccine PPV23. Trimethoprim-sulfamethoxazole and macrolides were the less active antibiotics. Three patients had non-bacteremic pneumococcal pneumonia, and two of them died.

**Conclusions:**

Pneumococcal colonization in SOTR is low with the most colonizing serotypes not included in the pneumococcal vaccines.

**Electronic supplementary material:**

The online version of this article (10.1186/s12879-019-4321-8) contains supplementary material, which is available to authorized users.

## Background

*Streptococcus pneumoniae* is one of the major causes of death worldwide [1, 2]. Invasive pneumococcal disease (IPD) is an important cause of disease in the elderly and young children (< 1 year), with an incidence rate of 13.8 and 11.3 cases per 100,000 population, respectively [1]. Other medical conditions such as transplantation also increase the risk of IPD [3–5].

In solid organ transplant (SOTR), *S. pneumoniae* can be a significant reason for morbidity and mortality, including non-invasive and invasive disease, with a 12.8-fold greater incidence of IPD compared to the general population [4]. Specific incidences vary according to the organ transplanted, with an incidence in kidney, lung, and liver transplant recipients of 1.04, 2.39, and 3.54 per 1000 transplant recipients/year [4]. Mortality, although not significant, has been higher in SOT recipients compared with non-immunosuppressed patients (28.6% vs. 14.1%) [4, 6].

Among the virulence factors of *S. pneumoniae*, polysaccharide capsule is one of the most important. Approximately ninety different pneumococcal serotypes have been identified depending on the chemical and antigenic differences of the capsule [7], twenty of which had been described as causing most IPDs [8, 9]. The awareness of which serotypes are the most invasive has allowed the development of current vaccines [10]. In SOTR, recommendations emphasize the importance of vaccinating all candidates before transplantation [11, 12]; however, this goal is still far from reality [13, 14]. A systematic review published by Eckerle et al. [15] in 2013 demonstrated that recommendations for the vaccination of SOTR are based on evidence from studies in healthy persons, with an urgent need to conduct vaccination trials in well-defined SOTR cohorts.

The colonization of the nasopharynx is an initial step for the evolution of IPD with transmission from human carriers [16]. Again, pneumococcal colonization has been mostly studied in children [17–19]. In healthy adults, the data about *S. pneumoniae* nasopharyngeal carriage is limited, with reported colonization rates of 4–13% [18, 20]. Information addressing *S. pneumoniae* colonization in the immunosuppressed population is scarce. Despite the significance of IPD in the transplant setting, no studies address pneumococcal colonization in SOT recipients. This information would be very useful to help understand the dynamics of serotypes in this population and learn if these serotypes are included in the vaccines currently used. The information available on serotype distribution consists only of a series of cases of IPD [4, 21–23], and vaccine recommendations have been made extrapolating data from healthy children.

In this study, we describe the rate of *S. pneumoniae* colonization in SOTR in two different seasonal periods, the capsular serotypes and the antimicrobial susceptibility/ resistance pattern.

## Methods

### Study design

A prospective observational cohort of SOTR was held at the University Hospital Virgen del Rocío, Seville, Spain. All patients included met the following inclusion criteria: a) adult patients (≥16 years); b) SOT recipients conducting their monitoring visits at the study center during the period of inclusion; c) survival after the transplant longer than 7 days; and d) written informed consent. Patients were excluded if they did not attend regular outpatient follow-up. A sample of 500 patients was included, expecting 20% of nasopharyngeal pneumococcal carriage, as in other immunosuppressed patients [3, 20, 24].

All patients were attended to in the outpatients’ clinic twice: at winter (December 2014–February 2015) and six months thereafter, spring/summer (June–August 2015). In each of these dates, structured interviews, as well as microbiological studies, were carried out. Clinical data of each patient regarding demographics, household contacts, contact with pneumococcal vaccinated and unvaccinated children, previous pneumococcal and influenza vaccination, transplant-related variables such as organ type, time from the transplantation, immunosuppression regimens and prior rejection, as well as chronic co-morbidities, antibiotic use in the last three months, prior hospitalization in the previous 30 days, and respiratory symptoms were obtained.

In cases of pneumococcal disease, all needed extra visits were performed, and clinical signs and symptoms, biochemical analysis, chest X-ray findings, antiviral and antibacterial therapies, concomitant or secondary infections, and outcomes, including mortality, were recorded.

### Sampling and isolation

Two different combined nasopharyngeal swab samples were obtained from each patient in both winter and spring/summer visits to increase detection rate [25, 26]. Samples were obtained by using a sterile transport swab (EO36-REV.00, COPAN, Brescia, Italy), and swabs were placed in a tube with 1.0 ml of AMIES transport medium according to the pneumococcal carriage studies protocol of the World Health Organization [25, 26]. The samples were cultured on Columbia Blood agar in 5% CO_2_ at 37 °C, and the α-hemolytic colonies were tested for optochin susceptibility and bile solubility [27]. Pneumococcal isolates were stored at − 80 °C in skimmed milk until further analysis.

### Antimicrobial susceptibility studies

Minimum inhibitory concentrations (MICs, mg/l) of levofloxacin, trimethoprim-sulfamethoxazole, penicillin, amoxicillin, cefotaxime, ceftriaxone, erythromycin, azithromycin and vancomycin for each *S. pneumoniae* isolate were determined using E-test strips [28]. MIC results were interpreted for all the antibiotics according to the Clinical & Laboratory Standards Institute CLSI, [29] breakpoint. Studies were performed in duplicate.

### Capsular typing

Isolates were typed using the modified scheme of sequential multiplex PCR protocol with sequential reactions described previously [30–32]. The serotypes studied were: 1, 2, 3, 4, 5, 6A/B, 6C/D, 7C/B/40, 7F, 8, 9 N/L, 9 V/A, 10A, 11A/D, 12F/A, 14, 15A, 15B/C, 16F, 17F, 18C/A/B/F, 19A, 19F, 20, 22F/A, 23A, 23B, 23F, 31, 33F, 34, 35B, 35F/47F, 37 and 38/25F. Briefly, each of the reactions included four serotype-specific primer pairs and a conserved region of the cps operon as internal positive control.

### Statistical analysis

Data obtained in the study was analysed using the SPSS statistical software (version 24.0, SPSS Inc., Chicago, Illinois). A descriptive analysis of all data was performed. The chi-square test was used for categorical variables and the t-Student for continuous variables, when appropriate. A multivariate logistic regression analysis of factors potentially associated with pneumococcal nasopharyngeal carriage and disease was performed, including significant variables in the bivariate analyses and clinically relevant variables. Statistical significance was established at *P* < 0.05.

## Results

### Characteristics of the study population

Five hundred SOT recipients were included in the study: 353 (70.6%) were kidney recipients, 106 (21.2%) liver recipients and 39 (7.9%) heart recipients. Most patients received tacrolimus, mycophenolate and steroids as immunosuppressant therapy. Twenty-four percent of patients reported having received pneumococcal vaccination. Clinical and demographic data are shown in Table 1. In winter 500 samples were collected, while in spring/summer 461 samples were. The difference in samples collection was due to 39 patients (7.8%) not attending to the spring/summer visit.

**Table 1:** Baseline characteristics and outcome of patients according to their pneumococcus colonization status

	Total *n* = 500	Not colonized *n* = 461	Colonized *n* = 39	*P* OR (CI95%)
Sex (male) - n (%)	322 (64.4)	291 (63.1)	31 (79.5)	<0.05 (0.20–0.98)
Age years, median (range)	54.4 (45–64)	54.5 (45–64)	53.8 (42–64)	ns
Type of transplant- n (%)				
• Kidney	353 (70.6)	324 (70.3)	29 (74.4)	ns
• Liver	106 (21.2)	100 (21.7)	6 (15.4)	
• Heart	39 (7.8)	35 (7.6)	4 (10.3)	
• Liver-kidney	2 (0.4)	2 (0.4)	0 (0)	
Time from transplant, median (range)				
• 0–180 days	94 (18.8)	88 (19.1)	6 (15.4)	ns
• >180–360 days	34 (6.8)	31 (6.7)	3 (7.7)	
• >1–5 years	139 (27.8)	131 (28.4)	8 (20.5)	
• > 5 years	233 (46.6)	211 (45.8)	22 (56.4)	
Charlson Comorbidity Index				
• 0 point	110 (22)	103 (22.3)	7 (17.9)	ns
• 1 points	94 (18.8)	89 (19.3)	5 (12.8)	
• 2 points	83 (16.6)	76 (16.5)	7 (17.9)	
• ≥3 puntos	213 (42.6)	193 (41.9)	20 (51.3)	
Cohabiting children- n (%)	172 (34.4)	149 (32.3)	23 (59)	0.001 (1.55–5.87)
• Children pneumococcal vaccination	18 (3.6)	17 (4.3)	1 (2.8)	ns
Prior pneumococcal infection- n (%)	44 (8.8)	38 (8.2)	6 (15.4)	ns
Prior pneumococcal vaccine- n (%)	121 (24.2)	115 (24.9)	6 (15.4)	ns
Baseline immunosuppression- n (%)				ns
• Tacrolimus	409 (81.8)	375 (81.3)	34 (87.2)	
• Mycophenolate	377 (75.4)	347 (75.3)	30 (76.9)	
• Ciclosporine	58 (11.6)	55 (11.9)	3 (7.7)	
• mTOR	79 (15.8)	75 (16.3)	4 (10.3)	
• Azathyoprine	10 (2)	10 (2.2)	0 (0)	
• Glucocorticoids	326 (65.2)	304 (65.9)	22 (56.4)	
Thymoglobulin induction < 6 m- n (%)	28 (5.6)	26 (5.6)	2 (5.1)	ns
Prior organ rejection	77 (15.4)	70 (15.2)	7 (17.9)	ns
Outcomes: - n (%)				
• Pneumococcal infection	3	2 (0.4)	1 (2.5)	ns
• Death	9	9 (2)	0 (0)	
• Rejection	2	2 (0.4)	0 (0)	
• Graft loss	7	7 (1.6)	0 (0)	

Abbreviations: *OR* odds ratio, *CI* Confidence Interval, *mTOR* mammalian Target of Rapamycin, *ns* not significant

### Pneumococcal carriage and infection

Twenty-six patients (5.6%) were colonized in winter and 15 (3.2%) in spring/summer (*P* = 0.06). During the study period, three female patients (age range 53–71) were diagnosed with non-bacteremic pneumococcal pneumonia (Table 2); two of whom died.

**Table 2:** Non-bacteraemic pneumococcal pneumonia cases in SOT recipients

Cases of IPD	Case 1	Case 2	Case 3
Type of transplant	Kidney	Liver	Kidney
Time from transplant	0–6 months	> 5 years	> 5 years
Comorbidity	Breast and renal cancer	Chronic renal disease, hypogammaglobulinemia, and CMV infection	Flu infection, Chronic hepatic and renal diseases, hypertrophic cardiomyopathy
Prior pneumococcal vaccine	No	No	No
Cohabiting children (children)	Yes (not vaccinated)	Yes (not vaccinated)	No
Microbiological identification	Urinary antigen test	Bronchoalveolar lavage culture^a^	Urinary antigen test
Days of hospitalization	4 days (general ward)	17 days (ICU)^b^	9 days (ICU)
Previous colonization	No	No	Yes
Final result	Cured	Dead	Dead

^a^No capsular serotype identified; penicillin susceptible - ^b^ICU, intensive care unit

The overall pneumococcal colonization rate in the 961 samples obtained was 4%. There were two patients with pneumococcal colonization in both studied periods. Colonized SOT recipients compared to non-colonized patients were more frequently men (79.5% vs. 63.1%, *P* < 0.05) and cohabitated regularly with children (59% vs. 32.2%, *P* < 0.001). In multivariable analysis these factors were also related to pneumococcal carriage: sex (men) OR 0.44 (CI95% 0.20–0.98) and cohabiting with children OR 2.90 (CI95% 1.5–5.77) (Table 3). There was no relationship between pneumococcal carriage and the immunosuppressant agents used, type of transplant or time since the transplant, pointing out that the immunosuppression was not related to the colonization status (Table 1).

**Table 3:** Multivariable analysis: factors potentially related to pneumococcal nasopharyngeal carriage in solid organ transplant recipients

Variable	OR (CI95%)	*p*
Sex (male vs. female)	0.44 (0.20–0.98)	**0.046**
Cohabiting children (yes vs. no)	2.95 (1.50–5.77)	**0.002**
Prior pneumococcal vaccination (yes vs. no)	0.59 (0.24–1.45)	0.247

Bold data are significant

Abbreviations: *OR* odds ratio, *CI* Confidence Interval

Six colonized patients (15.4%) and 115 non-colonized patients (24.9%) had received pneumococcal vaccine prior to their inclusion (*P* < 0.0.5). At the end of the study follow-up, 10 patients (2%) died; 7 (1.4%) lost the graft and 2 (0.4%) suffered an organ rejection. Colonized and non-colonized SOT recipients had similar outcomes regarding the incidence of pneumococcal disease, rejection, as well as graft and patients’ survival (Table 1).

### Characteristics of pneumococcal isolates

**Fig. 1:**
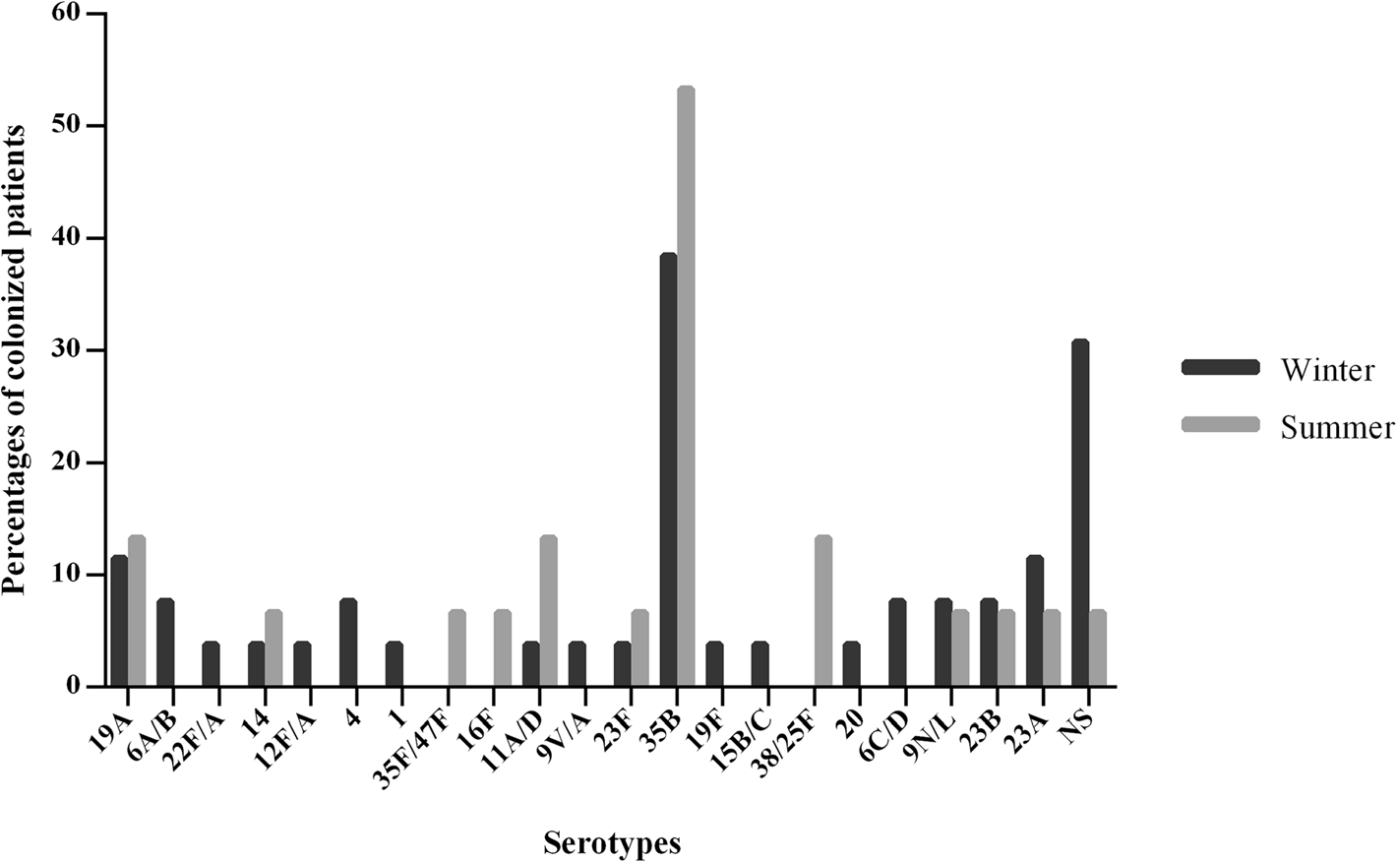
Serotypes seasonal distribution of the identified pneumococcal isolates. NS: No capsular serotypes

During the winter period, 26 different serotypes were identified. The most prevalent serotypes were (in decreasing order): 35B (38.5%), 19A and 23A (11.5%), 6A/6B, 4, 6C/D, 9 N/L and 23B (7.7% each) (Fig. 1). Overall, 52.7% of the winter isolates were included PCV23, while 47.2% were not included. Moreover, in 8 of the winter patients (30.8%) with pneumococcal isolates, no serotypes were identified by the molecular method used.

Sixteen isolates were resistant to azithromycin and erythromycin (36.36%), 15 isolates to clarithromycin (34.09%), 14 isolates to trimethoprim-sulfamethoxazole (31.82%), 2 isolates to oral penicillin (18.18%), and none to intravenous penicillin, amoxicillin, cefotaxime, ceftriaxone, levofloxacin and vancomycin (See Additional file 1: Table S1).

In the spring/summer period, 15 different serotypes were identified, 35B (53.3%), 19A, 11A/D, and 38/25F (13.3%) (In decreasing order) being the most prevalent ones (Fig. 1). Moreover, 33.3% of the spring/summer isolates were included in PPV23, while 66.6% were not included. The serotype of a patient of the spring/summer period (6.7%) could not be identified with the molecular method used.

A reduction in the rate of antimicrobial resistance was observed in this period. Six isolates were resistant to trimethoprim-sulfamethoxazole (27.27%), 4 isolates to oral penicillin, azithromycin, erythromycin and clarithromycin (18.18%), and 2 isolates to amoxicillin (9.09%), and as in the winter period no isolate was resistant to intravenous penicillin, cefotaxime, ceftriaxone, levofloxacin and vancomycin (See Additional file 2: Table S2).

The multiple-serotype carriage was similar in both study periods: 53.8 and 46.6%, respectively. Two patients were colonized during both periods with the capsular serotype 19A, and one of them was also colonized with the capsular 35B serotype in both periods.

Taking into account all the pneumococcal isolates collected in the study (*n* = 57), the most frequent capsular serotypes were 35B (31.6%), 19A (8.77%) and 23A (7.02%). Twenty-six (45.6%) serotypes were included in any of the pneumococcal vaccines (PCV13 and PPV23), while 31 (54.4%) serotypes were not included.

On the basis of CLSI breakpoints, the percentages of resistant isolates were 30.3% for trimethoprim-sulfamethoxazole, azithromycin and erythromycin, 28.79% for clarithromycin, 9.09 and 3.03% for oral penicillin and amoxicillin, respectively. No resistance was observed in the cases of intravenous penicillin, cefotaxime, ceftriaxone, levofloxacin, and vancomycin (Table 4).

**Table 4:** In vitro activities of 11 antibiotics against *S. pneumoniae* clinical isolates from SOT recipients

Antibiotic	CLSI breakpoints^a^ (*%* of isolates)			PK/PD	
	Susceptible	Intermediate	Resistant	Susceptibility (% of isolates)	Breakpoint (μg/ml)
O. Penicillin	39.39	51.52	9.09	39.39	≤0.06
I. Penicillin	100	0	0	100	≤2
Amoxicillin	87.88	9.09	3.03	87.88	≤2
Cefotaxime	100	0	0	100	≤1
Ceftriaxone	96.97	3.03	0	96.97	≤1
Azithromycin	53.03	16.67	30.30	53.03	≤0.5
Erythromycin	66.67	3.03	30.30	66.67	≤0.25
Clarithromycin	71.21	0	28.79	71.21	≤0.25
Levofloxacin	75.76	24.24	0	75.76	≤2
Vancomycin	100	0	0	100	≤1
Trimethoprim-sulfamethoxazole	53.03	16.67	30.30	53.03	≤0.5

Abbreviations: *O* oral, *I* intravenous

^a^The CLSI breakpoints used were ≤ 0.06 mg/L (susceptible), 0.12 to 1 mg/L (intermediate), and ≥ 2 mg/L (resistant) for oral penicillin and ≤ 2 mg/L (susceptible), 4 mg/L (intermediate), and ≥ 8 mg/L (resistant) for intravenous penicillin; ≤2 mg/L (susceptible), 4 mg/L (intermediate), and ≥ 8 mg/L (resistant) for amoxicillin; ≤1 mg/L (susceptible), 2 mg/L (intermediate), and ≥ 4 mg/L (resistant) for cefotaxime and ceftriaxone; ≤0.5 mg/L (susceptible), 1 mg/L (intermediate), and ≥ 2 mg/L (resistant) for azithromycin; ≤0.25 mg/L (susceptible), 0.5 mg/L (intermediate), and ≥ 1 mg/L (resistant) for erythromycin and clarithromycin; ≤2 mg/L (susceptible), 4 mg/L (intermediate), and ≥ 8 mg/L (resistant) for levofloxacin; ≤1 mg/L (susceptible) for vancomycin; and ≤ 0.5 mg/L (susceptible), 1–2 mg/L (intermediate), and ≥ 4 mg/L (resistant) for trimethoprim-sulfamethoxazole

## Discussion

To our knowledge this is the first study conducted to evaluate pneumococcal colonization in SOT recipients. In this study we found that colonization incidence is lower than reported in other immunosuppressed patients such as HIV-infected adults [20, 24] and has no relation with the seasonal period studied. Colonized SOTR were more frequently men who cohabited with children. Moreover, almost half of the serotypes found were not included in the vaccines.

In the present study, as reported for non-immunocompromised patients [33–36], the colonization rate was less than 5% of the SOT recipients. This rate is considerably lower than that observed in healthy children (up to 54%) [37, 38]. Indeed, there was no relationship between pneumococcal carriage and the immunosuppressant agents used, type of transplant or time since transplantation, pointing out that the immunosuppression was not related to the colonization status.

A seasonal effect trend on the rate of pneumococcal colonization in SOTR was observed. In accordance with it, Numminen et al. [39], in a cohort of 223 infants in Asia, in 2015, concluded that climate did affect seasonal pneumococcal transmission, which acted similarly across the studied geographical regions.

On the other hand, epidemiological exposure to pneumococci, as occurs when cohabiting with children, was associated with nasopharyngeal pneumococcal colonization in SOT recipients. A trend towards a higher incidence of pneumococcal nasopharyngeal carriage was also observed among non-vaccinated patients. An important issue to highlight is the low accomplishment of the recommendation of pneumococcal vaccination; only a fourth of patients had received it, which is significantly lower than that of other recommended vaccines such as influenza vaccination in the SOT population [40].

In one-fifth of the colonized patients, the serotype was non-tipable and a high serotype diversity, 21 serotypes, was observed, as reported in previous studies [36]. Of the most prevalent serotypes found, serotypes 35B and 23A are not included in PPV23 and PCV13 vaccines, while serotype 19A is included in both vaccines. Serotype 35B has been reported as one of the most common serotypes in non-immunocompromised children and adults [41–44]. In the present study, 54.4% of serotypes colonizing SOTR were not included in current vaccines (PPV23 and PCV13). The finding of non-vaccine serotypes colonizing SOTR is a fact to be assessed given that it represents the previous step to invasive disease in susceptible patients. Moreover, it allows us to study the potential invasiveness of the more unknown serotypes, which takes advantage of the ecological niche created by the disappearance of vaccine able serotypes. Unfortunately, serotypes causing pneumococcal pneumonia could not be obtained in patients in this study and could, therefore, not be compared with those found in the nasopharinx of healthy SOTR. Despite this, the theoretical vaccine-preventable proportion of cases of IPD, using 13-valent vaccine in 2010 one year after its license in Europe, is estimated at 73% [45]. The emergence of non-vaccine serotypes remains an important issue, and according to the European Centre for Disease Prevention and Control, continued monitoring in Europe is essential for assessing interventions and informing the development of new vaccines [1].

Few studies have analysed the serotypes of pneumococcus involved in SOTR pneumococcal diseases, most of them including patients with IPD. A study carried out in 2013 in Spain by Lujan et al. [3] concluded that the serotypes 10A, 11A, and 33F, not included in the PCV13 vaccine, were the most frequently isolated in immunocompromised patients with IPD although their prevalence was low [3]. Kumar et al. [4] reported that the most frequent serotypes in SOT recipients with IPD were 23F, 22F, and 19F. They reported that only one-fourth of the SOT recipients with IPD had been previously vaccinated with PPV23 vaccine, and 65 and 85% of SOT recipients suffered an invasive pneumococcal disease caused by serotypes included in the PCV13 and PPV23 vaccines, respectively [4].

One-third of the isolates during the winter season were resistant to macrolides and cotrimoxazole, and 11% were resistant to quinolones. This proportion halved during the spring-summer season. Notably, the most prevalent serotype found (35B) presented 38% of resistance to macrolides and trimethoprim-sulfamethoxazole. Beall et al. [46], in 2002, also reported the high incidence of invasive serotype 35B in the US. In that study, 68 isolates (69%) were non-susceptible to penicillin; all of them with decreased susceptibility or non-susceptibility to cefotaxime and 11 isolates (16%) were resistant to trimethoprim-sulfamethoxazole. Moreover, in the present study, one penicillin-resistant isolate was highly resistant to cefotaxime (MIC, 8 μg/ml) and resistant to trimethoprim-sulfamethoxazole, tetracycline, erythromycin, clindamycin, and chloramphenicol. It is still not defined whether the resistance of certain serotypes to penicillin is due to innate biological features or the high usage of β-lactams antibiotics [46]. A higher resistance of the serotype 35B has been reported recently from isolates causing IPD in children, with 91% of the isolates penicillin non-susceptible, 46.2% erythromycin resistant, 21.8% trimethoprim-sulfamethoxazole resistant, and 16.7% were considered multidrug-resistant [42].

There were three cases of non-bacteraemia pneumococcal pneumonia during the study, only one of them previously colonized. However, two out of the three patients died, both with important comorbidities. None of these patients was previously vaccinated against pneumococcus. The absence of previous vaccination in SOTR had been linked to unfavourable IPD outcomes [46]. Although the number of cases with non-bacteraemia pneumococcal pneumonia was too low in our study to establish robust conclusions, we consider that the unfavourable outcome strengthens the recommendation for pneumococcal vaccination in SOTR [47].

We should point out some limitations of this study. First, it is possible that some mild cases of pneumococcal disease were not diagnosed; however, no IPD cases were missed since all patients were carefully reviewed and guided to report any respiratory symptoms or fever during the study period. Secondly, the small number of cases of pneumococcal pneumonia did not permit to stablish a robust association with the lack of pneumococcal vaccination. Finally, there was no official record of pneumococcal vaccination; consequently, this information is missing for 22.4% of the patients.

## Conclusions

The present study shows that the rate of pneumococcal colonization in SOTR is low, similar to that reported in the general adult population. The incidence of non-bacteremic pneumococcal pneumonia was low, although two out of the three patients died, suggesting the need of pneumococcal vaccination for the SOTR.

## Additional files

Additional file 1: **Table S1**. In vitro activities of 11 antibiotics against *S. pneumoniae* clinical isolates from SOT recipients in winter (DOCX 31 kb)

Additional file 2: **Table S2**. In vitro activities of 11 antibiotics against *S. pneumoniae* clinical isolates from SOT recipients in summer (DOCX 18 kb)

## Data Availability

The data used and/or analysed during the study are on the manuscript, any other data and or analysis are available from the corresponding author.

## Abbreviations

CLSI: Clinical & Laboratory Standards Institute
IPD: Invasive pneumococcal disease
MICs: Minimum inhibitory concentrations
SOTR: Solid organ transplant recipient
