# Supplementary material for: Prevalence and clinical impact of Streptococcus pneumoniae nasopharyngeal carriage in solid organ transplant recipients
Source: BMC Infect Dis. 2019 Aug 6;19:697. doi: 10.1186/s12879-019-4321-8 (PMC6685160; doi:10.1186/s12879-019-4321-8)
Supplement: Supplementary file 1 — Table S1. In vitro activities of 11 antibiotics against S. pneumoniae clinical isolates from SOT recipients in winter (DOCX 31 kb) [file 12879_2019_4321_MOESM1_ESM.docx]

**Table S1** *In vitro* activities of 11 antibiotics against *S. pneumoniae* clinical isolates from SOT recipients in winter

| **Clinical isolated** | **MIC (mg/L) / (S,I,R)** | | | | | | | | | | | | | | | | | | | | |
| --- | --- | --- | --- | --- | --- | --- | --- | --- | --- | --- | --- | --- | --- | --- | --- | --- | --- | --- | --- | --- | --- |
|  | **O. PEN** | | **I. PEN** | | **AMX** | | **CTX** | | **CRO** | | **AZM** | | **ERI** | | **CLR** | | **LEV** | | **VAN** | | **SXT** |
| **HUVR**  **1** | 0.094 / (I) | 0.094 / (S) | | 0.023 / (S) | | 0.064 / (S) | | 0.023 / (S) | | 1 / (I) | | 0.5 / (I) | | 0.094 / (S) | | 4 / (I) | | 0.5 / (S) | | 0.064 / (S) | |
| **HUVR**  **39** | 0.016 / (S) | 0.016 / (S) | | 0.016 / (S) | | ≤ 0.016 / (S) | | 0.006 / (S) | | 0.5 / (S) | | 0.25 / (S) | | 0.094 / (S) | | 1 / (S) | | 0.5 / (S) | | 0.19 / (S) | |
| **HUVR**  **79** | 1 / (I) | 1 / (S) | | 0.5 / (S) | | 0.38 / (S) | | 0.5 / (S) | | 0.75 / (I) | | 0.094 / (S) | | 0.125 / (S) | | 1.5 / (S) | | 0.75 / (S) | | 8 / (R) | |
| **HUVR**  **80** | 0.023 / (S) | 0.023 / (S) | | < 0.016 / (S) | | 0.023 / (S) | | 0.008 / (S) | | 0.252 / (S) | | 0.125 / (S) | | 0.094 / (S) | | 2 / (S) | | 0.38 / (S) | | 0.5 / (S) | |
| **HUVR 137** | 1.5 / (I) | 1.5 / (S) | | 1.5 / (S) | | 0.38 / (S) | | 0.25 / (S) | | 0.38 / (S) | | 0.19 / (S) | | 0.094 / (S) | | 2 / (S) | | 0.75 / (S) | | 4 / (R) | |
| **HUVR 167** | < 0.016 / (S) | < 0.016 / (S) | | 1 / (S) | | 0.125 / (S) | | 0.125 / (S) | | 0.125 / (S) | | 0.064 / (S) | | 0.125 / (S) | | 0.75 / (S) | | 0.75 / (S) | | 0.38 / (S) | |
| **HUVR 181** | 0.19 / (I) | 0.19 / (S) | | 0.19 / (S) | | 0.032 / (S) | | 0.016 / (S) | | 0.19 / (S) | | 0.125 / (S) | | 0.094 / (S) | | 2 / (S) | | 0.5 / (S) | | 3 / (I) | |
| **HUVR 196** | < 0.016 / (S) | < 0.016 / (S) | | <0.016 / (S) | | 0.032 / (S) | | 0.006 / (S) | | 0.38 / (S) | | 0.19 / (S) | | 0.094 / (S) | | 2 / (S) | | 0.75 / (S) | | 0.125 / (S) | |
| **HUVR 204** | 1 / (I) | 1 / (S) | | 1 / (S) | | 0.75 / (S) | | 0.5 / (S) | | 8 / (R) | | 16 / (R) | | 12 / (R) | | 3 / (I) | | 0.75 / (S) | | > 32 / (R) | |
| **HUVR 205** | 2 / (R) | 2 / (S) | | 3 / (I) | | 1 / (S) | | 0.5 / (S) | | >256 / (R) | | >256 / (R) | | >256 / (R) | | 1.5 / (S) | | 0.75 / (S) | | > 32 / (R) | |
| **HUVR 222** | 0.047 / (S) | 0.047 / (S) | | 0.125 / (S) | | 0.19 / (S) | | 0.125 / (S) | | >256 / (R) | | 8 / (R) | | 4 / (R) | | 1.5 / (S) | | 0.125 / (S) | | 4 / (R) | |
| **HUVR 272** | 1.5 / (I) | 1.5 / (S) | | 3 / (I) | | 1 / (S) | | 0.5 / (S) | | >256 / (R) | | >256 / (R ) | | >256 / (R) | | 2 / (S) | | 0.5 / (S) | | >32 / (R) | |
| **HUVR 303** | 0.25 / (I) | 0.25 / (S) | | 0.19 / (S) | | 0.064 / (S) | | 0.125 / (S) | | 0.5 / (S) | | 0.125 / (S) | | 0.064 / (S) | | 2 / (S) | | 0.5 / (S) | | >32 / (R) | |
| **HUVR 315** | 0.016 / (S) | 0.016 / (S) | | 0.016 / (S) | | <0.016 / (S) | | 0.004 / (S) | | 0.5 / (S) | | 0.094 / (S) | | 0.047 / (S) | | 1.5 / (S) | | 0.38 / (S) | | 0.5 / (S) | |
| **HUVR 340** | 0.5 / (I) | 0.5 / (S) | | 0.5 / (S) | | 0.094 / (S) | | 0.125 / (S) | | >256 / (R) | | >256 / (R) | | 0.38 / (S) | | 1.5 / (S) | | 0.25 / (S) | | 0.125 / (S) | |
| **HUVR 341** | 0.25 / (I) | 0.25 / (S) | | 0.125 / (S) | | 0.19 / (S) | | 0.094 / (S) | | >256 / (R) | | >256 / (R) | | 1 / (R) | | 2 / (S) | | 0.5 / (S) | | 0.19 / (S) | |
| **HUVR 343** | 0.125 / (I) | 0.125 / (S) | | 0.125 / (S) | | 0.47 / (S) | | 0.047 / (S) | | >256 / (R) | | >256 / (R) | | >256 / (R) | | 2 / (S) | | 0.75 / (S) | | 0.25 / (S) | |
| **HUVR 362** | <0.016 / (S) | <0.016 / (S) | | <0.016 / (S) | | <0.016 / (S) | | 0.002 / (S) | | 0.5 / (S) | | 0.19 / (S) | | 0.125 / (S) | | 0.5 / (S) | | 0.75 / (S) | | 0.19 / (S) | |
| **HUVR 368** | 0.25 / (I) | 0.25 / (S) | | 0.094 / (S) | | 0.094 / (S) | | 0.094 / (S) | | 0.5 / (S) | | 0.19 / (S) | | 0.064 / (S) | | 3 / (I) | | 0.5 / (S) | | 0.25 / (S) | |
| **HUVR 391** | 0.023 / (S) | 0.023 / (S) | | 0.016 / (S) | | 0.016 / (S) | | 0.012 / (S) | | 0.75 / (I) | | 0.25 / (S) | | 0.125 / (S) | | 2 / (S) | | 0.75 / (S) | | 0.25 / (S) | |
| **HUVR 426** | 0.016 / (S) | 0.016 / (S) | | <0.016 / (S) | | <0.016 / (S) | | 0.023 / (S) | | 0.38 / (S) | | 0.023 / (S) | | 0.125 / (S) | | 1.5 / (S) | | 0.5 / (S) | | 0.19 / (S) | |
| **HUVR 428** | 0.023 / (S) | 0.023 / (S) | | <0.016 / (S) | | <0.016 / (S) | | 0.008 / (S) | | 0.125 / (S) | | 0.064 / (S) | | 0.064 / (S) | | 2 / (S) | | 0.75 / (S) | | 0.38 / (S) | |
| **HUVR 431** | <0.016 / (S) | <0.016 / (S) | | <0.016 / (S) | | <0.016 / (S) | | 0.006 / (S) | | 0.38 / (S) | | 0.094 / (S) | | 0.047 / (S) | | 1.5 / (S) | | 0.5 / (S) | | 1.5 / (I) | |
| **HUVR 446** | 0.016 / (S) | 0.016 / (S) | | <0.016 / (S) | | <0.016 / (S) | | 0.008 / (S) | | 0.19 / (S) | | 0.094 / (S) | | 0.094 / (S) | | 1.5 / (S) | | 0.5 / (S) | | 0.094 / (S) | |
| **HUVR 462** | 0.75 / (I) | 0.75 / (S) | | 0.5 / (S) | | 0.38 / (S) | | 0.38 / (S) | | >256 / (R) | | 6 / (R) | | 1.5 / (R) | | 1 / (S) | | 0.5 / (S) | | >32 / (R) | |
| **HUVR 472** | 0.19 / (I) | 0.19 / (S) | | 0.125 / (S) | | 0.125 / (S) | | 0.094 / (S) | | 0.19 / (S) | | 0.094 / (S) | | 0.047 / (S) | | 4 / (I) | | 0.19 / (S) | | 0.064 / (S) | |

S: Susceptible; I: Intermediate; R: Resistant

^a^The CLSI breakpoints used were ≤0.06 mg/L (susceptible), 0.12 to 1 mg/L (intermediate), and ≥2 mg/L (resistant) for oral penicillin (O.PEN) and ≤2 mg/L (susceptible), 4 mg/L (intermediate), and ≥8 mg/L (resistant) for intravenous penicillin (I.PEN); ≤2 mg/L (susceptible), 4 mg/L (intermediate), and ≥8 mg/L (resistant) for amoxicillin (AMX); ≤1 mg/L (susceptible), 2 mg/L (intermediate), and ≥4 mg/L (resistant) for cefotaxime (CTX) and ceftriaxone (CRO); ≤0.5 mg/L (susceptible), 1 mg/L (intermediate), and ≥2 mg/L (resistant) for azithromycin (AZM); ≤0.25 mg/L (susceptible), 0.5 mg/L (intermediate), and ≥1 mg/L (resistant) for erythromycin (ERI) and clarithromycin (CLR); ≤2 mg/L (susceptible), 4 mg/L (intermediate), and ≥8 mg/L (resistant) for levofloxacin (LEV); ≤1 mg/L (susceptible) for vancomycin (VAN); and ≤0.5 mg/L (susceptible), 1-2 mg/L (intermediate), and ≥4 mg/L (resistant) for trimethoprim-sulfamethoxazole (SXT).
