# Supplementary material for: Prevalence and clinical impact of Streptococcus pneumoniae nasopharyngeal carriage in solid organ transplant recipients
Source: BMC Infect Dis. 2019 Aug 6;19:697. doi: 10.1186/s12879-019-4321-8 (PMC6685160; doi:10.1186/s12879-019-4321-8)
Supplement: Supplementary file 2 — Table S2. In vitro activities of 11 antibiotics against S. pneumoniae clinical isolates from SOT recipients in summer (DOCX 18 kb) [file 12879_2019_4321_MOESM2_ESM.docx]

**Table S2** *In vitro* activities of 11 antibiotics against *S. pneumoniae* clinical isolates from SOT recipients in summer

| **Clinical isolated** | **MIC (mg/L) / (R, I, S)** | | | | | | | | | | |
| --- | --- | --- | --- | --- | --- | --- | --- | --- | --- | --- | --- |
|  | **O. PEN** | **I. PEN** | **AMX** | **CTX** | **CRO** | **AZM** | **ERI** | **CLR** | **LEV** | **VAN** | **SXT** |
| **HUVR 260** | 1 / (I) | 1 / (S) | 2 / (S) | 0.5 / (S) | 0.38 / (S) | 0.5 / (S) | 0.125 / (S) | 0.064 / (S) | 3 / (I) | 0.75 / (S) | 4 / (R) |
| **HUVR 446** | 0.023 / (S) | 0.023 / (S) | <0.016 / (S) | <0.016 / (S) | 0.012 / (S) | 0.25 / (S) | 0.19 / (S) | 0.064 / (S) | 2 / (S) | 0.38 / (S) | 0.032 / (S) |
| **HUVR 489** | 1 / (I) | 1 / (S) | 2 / (S) | 0.5 / (S) | 0.38 / (S) | 0.25 / (S) | 0.125 / (S) | 0.094 / (S) | 1.5 / (S) | 0.5 / (S) | 8 / (R) |
| **HUVR 412** | <0.016 / (S) | <0.016 / (S) | <0.016 / (S) | <0.016 / (S) | 0.25 / (S) | 0.5 / (S) | 0.19 / (S) | 0.064 / (S) | 2 / (S) | 1 / (S) | 1.5 / (I) |
| **HUVR 414** | 0.023 / (S) | 0.023 / (S) | <0.016 / (S) | <0.016 / (S) | 0.008 / (S) | 0.19 / (S) | 0.047 / (S) | 0.032 / (S) | 1.5 / (S) | 0.5 / (S) | 0.25 / (S) |
| **HUVR 113** | <0.016 / (S) | <0.016 / (S) | <0.016 / (S) | <0.016 / (S) | 0.004 / (S) | 0.125 / (S) | 0.25 / (S) | <0.016 / (S) | 1.5 / (S) | 0.38 / (S) | 0.75 / (I) |
| **HUVR 129** | 0.016 / (S) | 0.016/ (S) | <0.016 / (S) | 0.016 / (S) | 0.008 / (S) | 0.25 / (S) | 0.094 / (S) | 0.064 / (S) | 2 / (S) | 0.38 / (S) | 0.38 / (S) |
| **HUVR 494** | 0.5 / (I) | 0.5 / (S) | 0.75 / (S) | 0.38 / (S) | 0.38 / (S) | 0.125 / (S) | 0.047 / (S) | 0.064 / (S) | 3 / (I) | 0.75 / (S) | 0.19 / (S) |
| **HUVR 166** | 0.016 / (S) | 0.016 / (S) | <0.016 / (S) | <0.016 / (S) | 0.008 / (S) | 0.38 / (S) | 0.125 / (S) | 0.094 / (S) | 3 / (I) | 0.38 / (S) | 0.125 / (S) |
| **HUVR 205** | 2 / (R) | 2 / (S) | 4 / (I) | 1 / (S) | 0.75 / (S) | >256 / (R) | >256 / (R) | >256 / (R) | 1.5 / (S) | 0.5 / (S) | >32 / (R) |
| **HUVR**  **44** | 0.19 / (I) | 0.19 / (S) | 0.19 / (S) | 0.19 / (S) | 0.64 / (S) | 12 / (R) | 3 / (R) | 2 / (R) | 3 / (I) | 0.75 / (S) | 1 / (I) |
| **HUVR 215** | 1.5 / (I) | 1.5 / (S) | 0.125 / (S) | 0.023 / (S) | 0.064 / (S) | 0.75 / (I) | 0.19 / (S) | 0.094 / (S) | 3 / (I) | 0.5 / (S) | 0.047 / (S) |
| **HUVR 309** | 0.032 / (S) | 0.032 / (S) | 0.125 / (S) | <0.016 / (S) | 0.006 / (S) | 0.75 / (I) | 0.25 / (S) | 0.064 / (S) | 3 / (I) | 0.38 / (S) | 0.25 / (S) |
| **HUVR 403** | 0.125 / (I) | 0.125 / (S) | 0.023 / (S) | 0.032 / (S) | 0.032 / (S) | 0.5 / (S) | 0.25 / (S) | 0.094 / (S) | 1.5 / (S) | 0.75 / (S) | 1.5 / (I) |
| **HUVR 258** | 2 / (R) | 2 / (S) | >256 / (R) | 1 / (S) | 1.5 / (I) | 1.5 / (I) | 0.25 / (S) | 0.125 / (S) | 1.5 / (S) | 0.5 / (S) | 3 / (I) |

S: Susceptible; I: Intermediate; R: Resistant

^a^The CLSI breakpoints used were ≤0.06 mg/L (susceptible), 0.12 to 1 mg/L (intermediate), and ≥2 mg/L (resistant) for oral penicillin (O.PEN) and ≤2 mg/L (susceptible), 4 mg/L (intermediate), and ≥8 mg/L (resistant) for intravenous penicillin (I.PEN); ≤2 mg/L (susceptible), 4 mg/L (intermediate), and ≥8 mg/L (resistant) for amoxicillin (AMX); ≤1 mg/L (susceptible), 2 mg/L (intermediate), and ≥4 mg/L (resistant) for cefotaxime (CTX) and ceftriaxone (CRO); ≤0.5 mg/L (susceptible), 1 mg/L (intermediate), and ≥2 mg/L (resistant) for azithromycin (AZM); ≤0.25 mg/L (susceptible), 0.5 mg/L (intermediate), and ≥1 mg/L (resistant) for erythromycin (ERI) and clarithromycin (CLR); ≤2 mg/L (susceptible), 4 mg/L (intermediate), and ≥8 mg/L (resistant) for levofloxacin (LEV); ≤1 mg/L (susceptible) for vancomycin (VAN); and ≤0.5 mg/L (susceptible), 1-2 mg/L (intermediate), and ≥4 mg/L (resistant) for trimethoprim-sulfamethoxazole (SXT).
